# Comparative Performance of Four Established Neonatal Disease Scoring Systems in Predicting In-Hospital Mortality and the Potential Role of Thromboelastometry

**DOI:** 10.3390/diagnostics11111955

**Published:** 2021-10-21

**Authors:** Rozeta Sokou, Maroula Tritzali, Daniele Piovani, Aikaterini Konstantinidi, Andreas G. Tsantes, Georgios Ioakeimidis, Maria Lampridou, Stavroula Parastatidou, Nicoletta Iacovidou, Styliani Kokoris, Georgios K. Nikolopoulos, Petros Kopterides, Stefanos Bonovas, Argirios E. Tsantes

**Affiliations:** 1 Neonatal Intensive Care Unit, “Agios Panteleimon” General Hospital of Nikea, 3 D. Mantouvalou Str., Nikea, 18454 Piraeus, Greece; kmaronia@gmail.com (A.K.); giorgos.ioakeimidis@gmail.com (G.I.); litsamaria@hotmail.com (M.L.); stavroula.parastatidou@gmail.com (S.P.); 2 First Department of Pediatrics, National and Kapodistrian University of Athens, “Aghia Sophia” Children’s Hospital, Thivon 1, Goudi, 11527 Athens, Greece; martri.mt@gmail.com; 3 Department of Biomedical Sciences, Humanitas University, via Rita Levi Montalcini 4, Pieve Emanuele, 20090 Milan, Italy; dpiovani@hotmail.com (D.P.); sbonovas@gmail.com (S.B.); 4 IRCCS Humanitas Research Hospital, via Manzoni 56, Rozzano, 20089 Milan, Italy; 5 Laboratory of Haematology and Blood Bank Unit, “Attiko” Hospital, School of Medicine, National and Kapodistrian University of Athens, 1 Rimini Str., 12462 Athens, Greece; andreas.tsantes@yahoo.com (A.G.T.); stelkok19@gmail.com (S.K.); atsantes@yahoo.com (A.E.T.); 6 Neonatal Department, National and Kapodistrian University of Athens, Aretaieio Hospital, 15772 Athens, Greece; niciac58@gmail.com; 7 Medical School, University of Cyprus, Nicosia 1678, Cyprus; gknikolopoulos@gmail.com; 8 Intensive Care Unit, Excela Health Westmoreland Hospital, Greensburg, PA 15601, USA; pkopterides@gmail.com

**Keywords:** thromboelastometry, prognosis, neonatal severity score, coagulopathy

## Abstract

Background: To compare the prognostic accuracy of the most commonly used indexes of mortality over time and evaluate the potential of adding thromboelastometry (ROTEM) results to these well-established clinical scores. Methods: The study population consisted of 473 consecutive term and preterm critically-ill neonates. On the first day of critical illness, modified Neonatal Multiple Organ Dysfunction (NEOMOD) scoring system, Score for Neonatal Acute Physiology (SNAP II), Perinatal extension of SNAP (SNAPPE), and SNAPPE II, were calculated and ROTEM standard extrinsically activated (EXTEM) assay was performed simultaneously. Time-to-event methodology for competing-risks was used to assess the performance of the aforementioned indexes in predicting in-hospital mortality over time. Time-dependent receiver operator characteristics curves for censored observation were compared across indexes. The addition of EXTEM parameters to each index was tested in terms of discrimination capacity. Results: The modified NEOMOD score performed similarly to SNAPPE. Both scores performed significantly better than SNAP II and SNAPPE II. Amplitude recorded at 10 min (A10) was the EXTEM parameter most strongly associated with mortality (A10 < 37 mm vs. ≥37 mm; sHR = 5.52; *p* < 0.001). Adding A10 to each index apparently increased the prognostic accuracy in the case of SNAP II and SNAPPE II. However, these increases did not reach statistical significance. Conclusion: Although the four existing indexes considered showed good to excellent prognostic capacity, modified NEOMOD and SNAPPE scores performed significantly better. Though larger studies are needed, adding A10 to well-established neonatal severity scores not including biomarkers of coagulopathy might improve their prediction of in-hospital mortality.

## 1. Introduction

Prompt identification of neonates with increased risk of morbidity and mortality in Neonatal Intensive Care Units (NICUs) may result in optimal patient management. Several scoring systems have been developed and established to evaluate the severity of the disease and predict the prognosis of ill neonates [1].

The most updated information on the neonate’s clinical state is necessary for the accurate prediction of the outcome, irrespective of the management strategy. Data depicting the patient’s progress through time provide a more precise prediction than the initial information collected during the first few hours of life [2]. Commonly used scores, like clinical risk index for babies (CRIB) and score for neonatal acute physiology (SNAP), fail to overcome this problem and are poor predictors of outcome, as they are solely calculated at 12 and 24 h, respectively. The Neonatal Multiple Organ Dysfunction (NEOMOD) scoring system is used for prognosis of mortality in neonates with multiple organ dysfunction syndrome (MODS) [3].

Although the mortality risk of neonates admitted to the NICU usually varies with gestational age (GA) and birth weight (BW), other perinatal factors, physiological parameters, and disease severity may also contribute to mortality [4].

Systemic inflammation leads to coagulation system activation, while at the same time, components of the haemostatic mechanism significantly affect inflammatory process [5]. Studies in the literature have underlined the significance of interleukin (IL)-6 in the activation of coagulation, while tissue necrosis factor-α (TNF-α) and IL-1 are key regulators of physiologic anticoagulation. Consequently, dysregulated activation of systemic coagulation [6] is involved in vascular thrombotic disease, and also plays a crucial role in the pathogenesis of microvascular inflammatory compromise and subsequent multiple organ failure [7].

Rotational thromboelastography (TEG) and thromboelastometry (ROTEM) are point-of-care tests estimating the dynamics of blood coagulation [8]. It appears that platelets are the only hemostatic parameter included in the existing neonatal disease severity score systems. Viscoelastic tests (VMs) represent promising methods for the evaluation of the hemostatic status of ill neonates. Recently, it was demonstrated, that a ROTEM hypocoagulable profile on disease onset is an independent risk factor for in-hospital mortality in neonatal critical illness [9].

Several risk scores are used in clinical practice to predict short-term mortality in critically-ill neonates; however, scant data exist concerning their comparative prognostic capacity. The primary aim of the study was to compare for the first time the performance of already established neonatal disease scoring systems (modified NEOMOD, SNAP II, SNAP with Perinatal extension (SNAPPE), SNAPPE II scores) in predicting in-hospital mortality over time. As a secondary aim, the addition of ROTEM parameters to each index was evaluated to assess whether this could further improve the scores’ prognostic accuracy.

## 2. Materials and Methods

**Figure 1:**
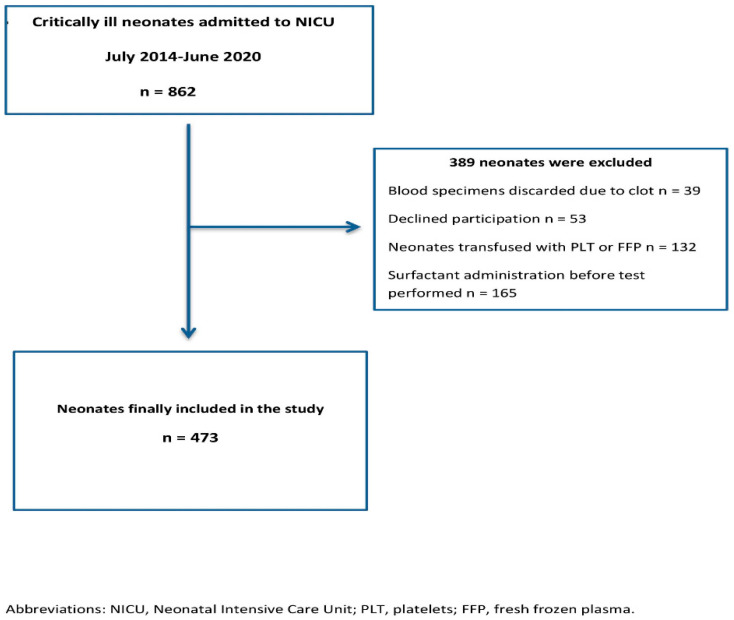
Flow-chart of study population.

The study population consists of consecutive term and preterm critically-ill neonates hospitalized in the NICU of General Hospital of Nikaia, Piraeus, Greece, over a period of 6 years (07/2014–06/2020). This study is an extension to a recently published trial estimating the prognostic role of ROTEM (Tem Innovations GmbH, Munich, Germany) in neonatal critical illness [9]. Recruitment data are presented in a flow diagram (Figure 1). 

The inclusion and exclusion criteria have been previously described [9]. Data on demographics, maternal and pregnancy history, maternal medications during pregnancy, neonatal physiological parameters, and clinical findings were recorded. On the first day of sepsis, suspected sepsis, perinatal hypoxia, and/or respiratory distress syndrome (RDS), arterial blood anticoagulated with 0.109 mol/L trisodium citrate (9:1, *v*/*v* blood anticoagulant) was analyzed on the ROTEM analyzer (Tem Innovations GmbH, Munich, Germany) using the extrinsically activated (EXTEM) thromboelastometry assay, as previously described [10,11]. The following EXTEM parameters were measured: Clotting Time (CT, seconds), defined as the time from test start until a clot firmness amplitude of 2 mm is reached; Clot Formation Time (CFT, seconds), indicating the time from the end of the CT until clot firmness amplitude of 20 mm is achieved; Alpha-angle (α°), the angle between the baseline and a tangent to the clotting curve through the 2 mm point; Clot firmness amplitude recorded at 10, 20, and 30 min (A10, A20, and A30); Maximum Clot Firmness (MCF, mm), representing the final strength of the clot; Lysis Index at 60 min (LI 60%), defined as the percentage of remaining clot in relation to the MCF over the 60-min observation period after CT; and Maximum Lysis (ML), which was observed during the run time, described as the percentage of MCF.

On the same day of ROTEM testing and prior to initiating antibiotic therapy, blood specimens for culture, routine biochemical tests, complete blood count, and C-reactive protein (CRP) were obtained. Chest radiograph, cerebrospinal fluid culture, and urine culture were performed whenever clinically indicated, as per our NICU protocol. Modified NEOMOD scoring system [12], SNAP II, SNAPPE II, and SNAPPE [13,14] were calculated at the same time with the EXTEM analysis. Data collection took place within the first 24 h after the disease onset. Clinical status was evaluated daily until discharge or death [15,16]. Each neonate included in the study was evaluated only once.

### Statistical Analysis

We present descriptive statistics of the baseline characteristics and ROTEM parameters as means ± standard deviations (SD), medians and interquartile ranges (IQR), or percentages when appropriate. 

In agreement with previous studies, in-hospital mortality was selected as the study outcome [13,17,18,19,20]. Time-to-event (survival) methodology for competing risks was used to assess how the four most frequently used indexes for mortality perform over time in predicting in-hospital death. Time to event was defined as the time from ROTEM analysis to the date of death or censoring (i.e., we censored data for patients still hospitalized in the NICU at the time of data extraction). Patient discharge was considered as a competing event for death [21], as it prevents the observation of the event of interest and is associated with it (i.e., the probability of dying at a certain time point is drastically lower in the group of patients discharged as compared to the group of patients still being followed in the NICU).

The cumulative incidence functions were estimated and plotted for the competing events of death and discharge. The association between the study outcome and the four prognostic indexes (SNAP II, SNAPPE II, SNAPPE, and modified NEOMOD scores, respectively) was quantified by means of Fine and Gray competing risk regression with robust estimators of the standard error and considering the sub-hazard function of the event of primary interest (death) [22]. The sub-hazard ratios (sHR) were presented with 95% confidence intervals (CI) and the respective *p*-values. 

Time-dependent receiver operating characteristic (ROC) curve analysis for censored observation, by means of inverse probability of censoring weighting, was used to evaluate the discrimination power of the four indexes in predicting patients who will die [23]. In time-dependent ROC-curve analysis, the status of an individual is observed and updated at each time point taking into account censored observations and competing events. Thus, the ROC curve can be constructed at several time points, and the discrimination power curve (i.e., the area under curve, AUC) can be compared during time, allowing a dynamic estimate of the performance of each individual index [23]. As a sensitivity analysis, we also calculated and plotted the prognostic performances of the four indexes in preterm (<37 weeks) and term (≥37 weeks) neonates.

A secondary aim of the study was to test the hypothesis that ROTEM variables could improve the prognostic power of indexes of mortality frequently used in clinical practice. In a very similar cohort of neonates, A10, CT, and MCF EXTEM ROTEM variables had been shown as significantly associated with mortality [9]. In the current study, A10 was the variable most strongly associated with mortality. We plotted the cumulative incidence curves for A10 dichotomized at the value of 37 mm, as previously reported [9], and compared them with the Gray’s test [24]. Then, we applied multivariable Fine and Gray regression, and calculated the sHR for death in models including SNAP II, SNAPPE II, SNAPPE, and modified NEOMOD, respectively, in addition to A10. We calculated and plotted the AUC over time for each one of these four multivariable models, and compared them with that of the corresponding univariable models, including the mortality indexes alone. The analyses were based on non-missing data (i.e., missing data were not imputed); less than 1% of observations were missing. For statistical analysis, we used Stata 15 (Stata Corp., College Station, TX, USA), and the R software. For all the tests, a two-tailed *p*-value < 0.05 was considered statistically significant.

## 3. Results

This cohort included 473 consecutive, critically-ill neonates. The population consisted predominantly of males (63.4%). The mean day of life of neonates’ enrolment was 10.0 days (SD = 19.8), while the median was 4 days (IQR: 2–10). The median gestational age was 37 weeks (IQR: 32–39), and the median BW was 2580 g (IQR: 1480–3240); 11% of the neonates (*n* = 52) were <3rd percentile of body weight; 8.5% (*n* = 40) of the study neonates were born with extremely low birth weight (<1000 g), where 17.3% (*n* = 82) were born with very low birth weight (1000 g–1500 g) and 22% (*n* = 104) were born with low birth weight (1500 g–2500 g). About half (*n* = 224; 47.4%) of the included neonates were preterm (<37 weeks of gestation). The 5 min APGAR score was <7 in 9.7% (*n* = 46) of the neonates. The most common comorbidities included respiratory distress syndrome (51.4%) and intraventricular hemorrhage of any grade (31.1%).

The other baseline characteristics, biochemical, ROTEM, and hematological measurements are presented in Table 1. 

**Figure 2:**
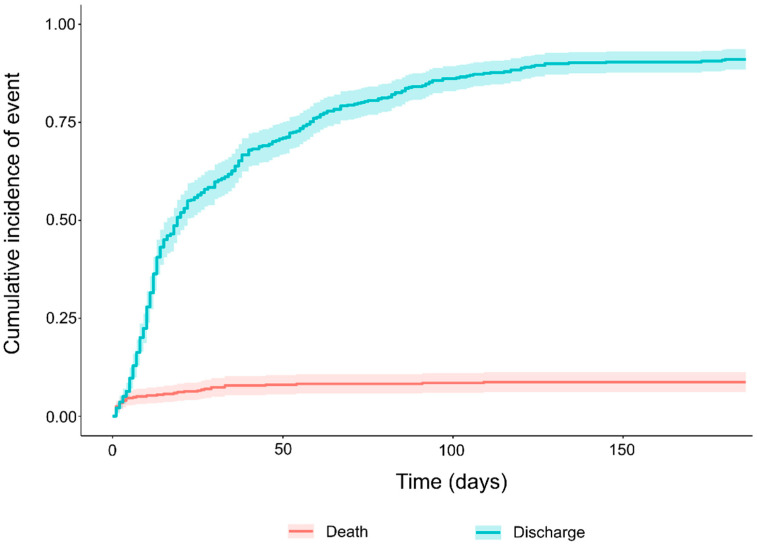
Overall cumulative probability of death.

Out of the 473 neonates, 42 (8.9%) died, 426 were discharged, and 6 were still in the NICU at data extractions (censored). The cumulative incidence curve showed an overall cumulative probability of death of 0.0871 (95% CI, 0.0831–0.0910) over an overall time at risk of 38.5 person-years (Figure 2).

### 3.1. Performance over Time of SNAP II, SNAPPE II, SNAPPE and Modified NEOMOD Score 

**Figure 3:**
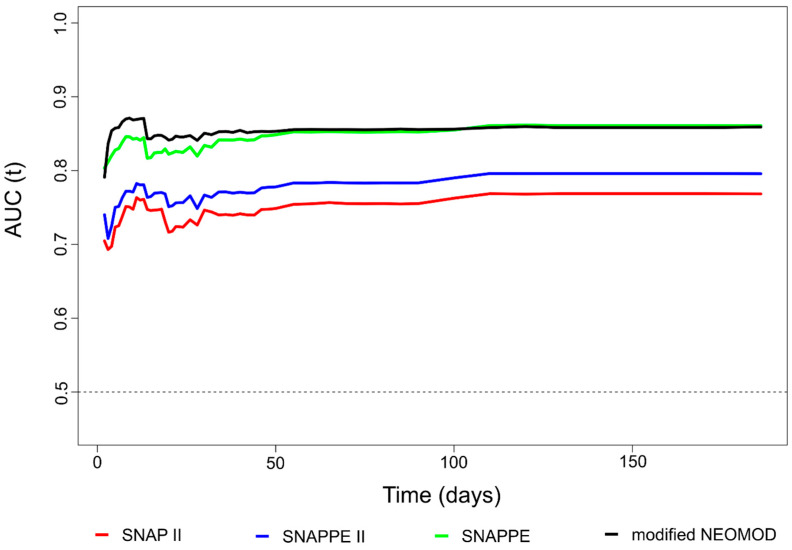
Over time prognostic performance of the four scores in predicting death.

The four ROC curves over time displaying the prognostic performance of SNAP II, SNAPPE II, SNAPPE, and the modified NEOMOD score in predicting death, are presented in Figure 3. 

The modified NEOMOD score performed similarly to SNAPPE with no statistically significant difference at any time point (Table 2). 

**Figure 4:**
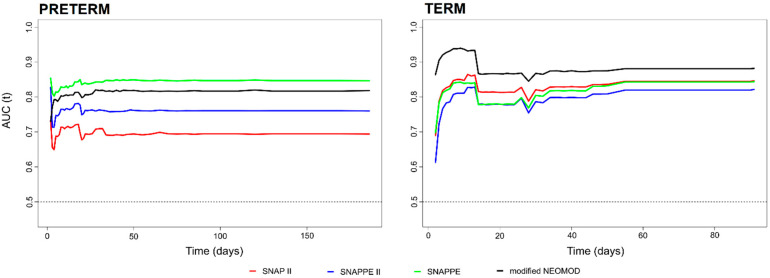
Performance of the four indexes in preterm and term neonates. SNAP II, red line; SNAPE II, blue line; SNAPPE, green line; modified NEOMOD, black line. The dotted line (of AUC = 0.5) means the model has no discrimination capacity.

Both the modified NEOMOD and the SNAPPE scores were significantly better than SNAP II and SNAPPE II at any time point besides at two weeks from study enrolment, when the difference did not reach significance (Table 2). The modified NEOMOD score was significantly better than SNAP II at any time point. As a sensitivity analysis, we assessed the performance of the indexes in preterm and term neonates (Figure 4). 

In preterm neonates, SNAPPE was apparently the best performing index; however, the differences with the modified NEOMOD score and SNAPPE II did not reach significance. The AUC over time of SNAPPE was significantly better than that of SNAP II at any time point besides the first week (Table 3).

In term neonates, the modified NEOMOD score was apparently the best performing index; however, it was significantly better than SNAP II and SNAPPE II in predicting exclusively short-term mortality (Figure 4 and Table 3). In fact, the difference in terms of AUC between the modified NEOMOD score and SNAPPE II was significant at days 1–13, while there was a statistically significant difference with SNAP II at days 1–10. We found no statistically significant difference regarding the prognostic power of the modified NEOMOD score and SNAPPE at any time point.

### 3.2. Multivariable Models including the A10 EXTEM Parameter

**Figure 5:**
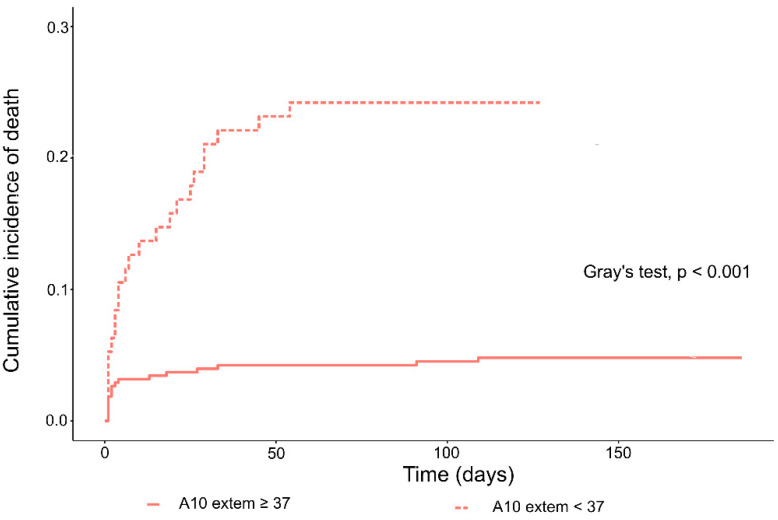
Clot amplitude at 10 min (A10): Plotted cumulative incidence of death.

A10 was the ROTEM parameter most strongly associated with mortality in Fine and Gray univariable regression (A10 < 37 mm vs. ≥37 mm; sHR = 5.52; 95% CI: 2.98–10.2; *p* < 0.001). The plotted cumulative incidence curves displayed an about five-fold higher risk of dying in the group of neonates showing values of A10 < 37 mm (Gray’s test *p* < 0.001; Figure 5).

The probability of death decreased by approximately 5.5% for each unit increase in the A10 parameter (sHR = 0.945; 95% CI: 0.928–0.962). Addition of the continuous version of the A10 parameter to each of the established prognostic indexes was the most convenient to increase the predictive power of our simple additive multivariable model. In the multivariable models including SNAP II together with A10 (sHR(SNAP II) = 1.060, *p* < 0.001; sHR(A10) = 0.960, *p* < 0.001), SNAPPE II together with A10 (sHR(SNAPPE II) = 1.051, *p* < 0.001; sHR(A10) = 0.966, *p* < 0.001), and SNAPPE together with A10 (sHR(SNAPPE) = 1.067, *p* < 0.001; sHR(A10) = 0.978, *p* = 0.044), both the original index and the ROTEM variable were independent, statistically significant predictors of mortality. This was not the case considering the model including the modified NEOMOD score together with A10 (sHR(NEOMOD) = 1.46, *p* < 0.001; sHR(A10) = 0.990, *p* = 0.49).

**Figure 6:**
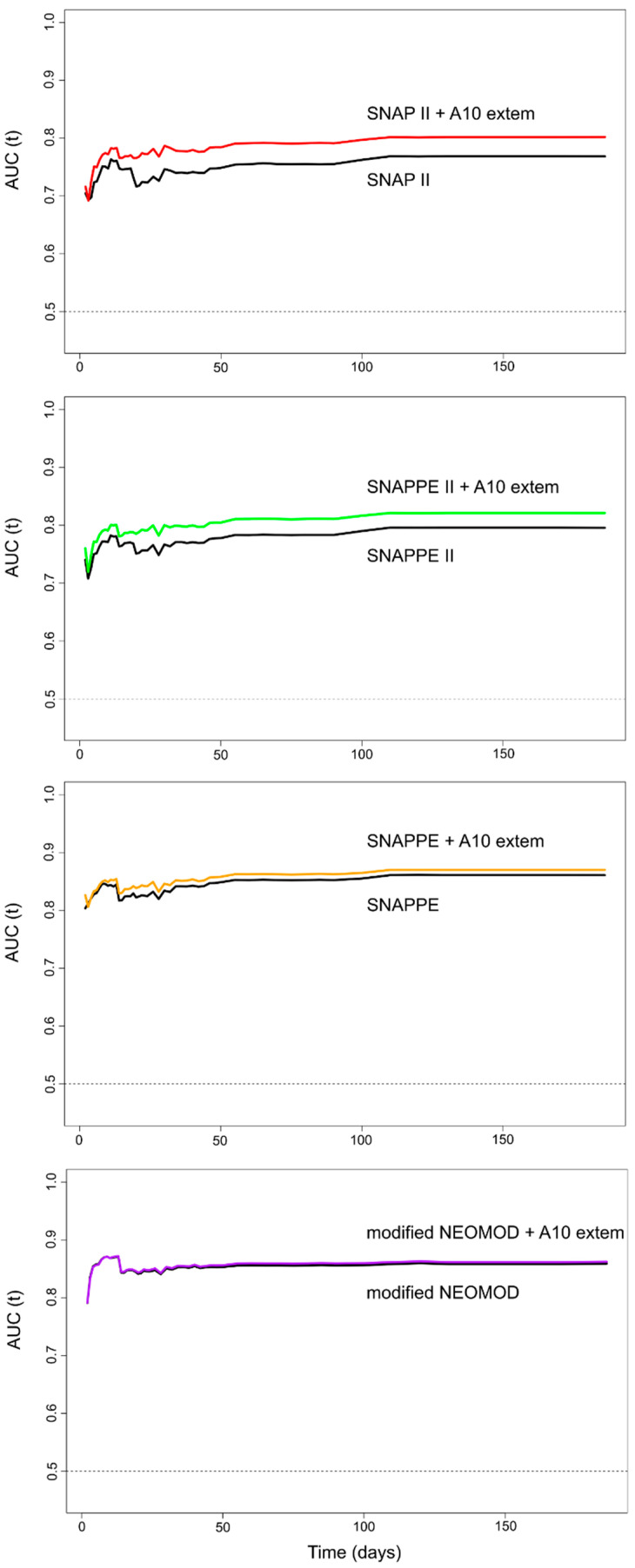
Over time prognostic performance of the scores in predicting death after the addition of A10. Red line, SNAP II + A10 extem; black line, SNAP II; green line, SNAPPE II + A10 extem; yellow line, SNAPPE + A10 extem; purple line, modified NEOMOD + A10 extem; the dotted line (of AUC = 0.5) means the model has no discrimination capacity.

We plotted the AUC over time of the four univariable models including the established indexes of mortality, and the four multivariable models after the addition of the continuous A10 parameter (Figure 6).

Apparently, the addition of A10 to the established index of mortality increased the prognostic power in the case of SNAP II and SNAPPE II. However, we observed no statistically significant difference between the AUC of any univariable model and the respective multivariable model including A10.

## 4. Discussion

To the best of our knowledge, this is the first study simultaneously assessing the time-dependent performance of four different prognostic indexes (SNAP II, SNAPPE II, SNAPPE, modified NEOMODs) in predicting mortality in critically-ill neonates over the entire hospital stay. In NICU-treated neonates, despite the changes in illness severity over time and several medical interventions, the risk of death still remains high during their hospitalization. Thus, the quantification of the time-dependent performance of these indexes might be of significant clinical value. The modified NEOMOD and the SNAPPE scores were the ones that performed best at almost any time point. Although SNAP II and SNAPPE II performed worse, especially in preterm infants, their use is very common since these indexes are much easier to calculate than SNAPPE. The addition of EXTEM A10 increased the prognostic power in the case of SNAP II and SNAPPE II, but without reaching statistical significance.

The use of prognostic indexes in NICUs has been established many years ago and they have been evaluated over time as useful tools to predict outcome, to quantify the initial risk of mortality and morbidity in critically-ill neonates along with stratifying them according to the risk level, to guide the optimal interventions, and to assess their effectiveness. These indexes have also served to compare lifetime outcomes in these vulnerable populations across hospitals or NICUs [2]. The SNAP, SNAPPE, and their next generation variants SNAP II and SNAPPE II are the most commonly used admission scores for the prediction of mortality risk in ill neonates. The original SNAP developed by Richardson et al. scores the worst physiologic derangements in each organ system in the first 24 h and involves 28 physiological parameters [25], whereas SNAPPE, the perinatal extension of SNAP by adding BW, small for gestational age (SGA), and five minute Apgar score, quantifies both physiological instability parameters and perinatal risk factors in one score [14]. However, these tools are labor-intensive and time-consuming, because of the large number of included components, while requiring up to 15 min for evaluation. Thus, Richardson et al. simplified them and created the SNAP II and SNAPPE II scores [13]. While the primary use of all these scores was to assess illness severity in the first 12–24 h of life, limited studies used them at later time points and for sequential measurements [26,27]. Griffin et al. found higher SNAP scores for up to 24 h before the clinical suspicion of sepsis [28]. Sundaram et al. concluded that neonates with severe septicemia are at a significantly higher risk of dying if they have high SNAP II score within the first 12 h from the onset of severe sepsis [29]. 

Taking into account the changes of clinical status of ill neonates over time and the effectiveness of clinical interventions, it seems rather precarious to evaluate illness severity based only on data collected during the first day of life. In line with this concept, in our study, the discriminative ability of SNAPPE, SNAP II, and SNAPPE II scores was assessed within the first 24 h of abrupt clinical deterioration of our subjects, with the SNAPPE showing significantly better prognostic performance than the other two scores (AUC point estimates: from 0.828 at 5 days to 0.862 at 120 days) at any time point, except on the 15th day of study enrolment when the difference did not reach significance. This finding was quite expected, as the SNAPPE score consists of an extensive list of objective laboratory variables in association with perinatal risk factors, such as five-minute Apgar score, BW, and SGA, and therefore more accurately describes illness severity. Additionally, SNAPPE II and SNAPPE showed better performance in preterm neonates when compared to SNAP II, probably because BW (included in SNAPPE and SNAPPE II) is the physiologic parameter with the major contribution to mortality rate in preterms [30,31], while all three scores had a similar performance in term neonates.

MODS is the most common cause of death for neonates admitted to NICUs. Janota et al. developed the NEOMOD score, which characterizes the severity of dysfunction in seven organ systems in very low birth weight (VLBW) neonates, and concluded that this score could evaluate the severity of MODS and predict mortality with accuracy [3,15]. As microvascular system derangement might be the earliest sign of MODS in neonates, Çetinkaya et al. developed the modified NEOMOD score, in which the microvascular system was added as the eighth system resulting in an extension of the current criteria of the NEOMOD scoring system [12]. 

In our study, the modified NEOMOD score showed better performance in predicting mortality overtime (AUC point estimates: from 0.858 at 5 days to 0.860 at 120 days) compared to SNAP-II and SNAPPE II scores. This finding is probably attributable to the fact that the modified NEOMOD score incorporates more clinical and laboratory parameters than the other two scores, possibly better reflecting the critically-ill neonate’s clinical status. In preterm neonates, the modified NEOMOD score showed performance similar to SNAP II, SNAPPE II, and SNAPPE scores at any time point. Additionally, the modified NEOMOD score could accurately predict mortality over time in term neonates (AUC point estimates: from 0.928 at 5 days to 0.881 at 120 days), with significantly better performance than SNAP II and SNAPPE II during the first five days after study enrollment. The prognostic power of the modified NEOMOD and SNAPPE scores was similar at any time point irrespective of gestational age. 

To our knowledge, this is the first study simultaneously assessing the discrimination ability of SNAP II, SNAPPE II, SNAPPE, and modified NEOMOD scores in predicting long-term in-hospital mortality in critically-ill neonates at disease onset. Lee et al. tested the time-dependent performance of the CRIB II in predicting mortality among VLBW neonates and reported that certain CRIB II cutoffs were significantly associated with time-dependent mortality, particularly within the first 90 days after birth [32]. In our study, CRIB II was not calculated as it is designed for use within the first hour of NICU admission [33]. 

Coagulation derangement often complicates the clinical course of critically-ill neonates and has the potential to be a life-threatening situation and increase mortality [34]. Thus, early and proper identification of hemostatic disorders is of great importance for the management of these patients. Among the laboratory tests used for diagnosing these disorders, global coagulation tests such as PT and APTT cannot provide a complete insight on patient’s hemostatic status. Contrarily, as VMs can detect and quantify dynamic changes in the hemostatic properties of a blood sample during the clot formation process, they could provide more specific information regarding the coagulation profile.

In two recent studies conducted by our research group using ROTEM, a hypocoagulable profile was detected in hypoxic and septic neonates, expressed with prolonged CT, CFT, and reduced A10 and MCF as compared to healthy neonates [10,11]. Recently, a hypocoagulable profile and impaired fibrinolysis on disease onset were identified as independent risk factors for in-hospital mortality in critically-ill neonates [9]. In the current study, A10 was the EXTEM parameter most strongly associated with mortality, as neonates showing values of A10 < 37 mm presented an about five-fold higher risk of in-hospital mortality. Adamzik et al. [35] and Ostrowski et al. [36] noted that a hypocoagulable profile on admission could predict mortality in adults with sepsis. The plausible mechanism underlying the association of coagulopathy with clinical outcome is probably the devastating host immune response leading to enhanced fibrin formation and its microvascular deposition, which results in subsequent organs dysfunction [37].

Neonatal coagulopathy and especially disseminated intravascular coagulation (DIC) are associated with worse prognosis in critically-ill neonates [34]. Although the hemostatic system has been increasingly recognized as a key player in the development and course of critical illness, except from platelets, no other biomarkers of coagulopathy are currently incorporated into the neonatal prognostic scores. Taking into account all the above information, we further examined the potential of increasing the prognostic power of each of the aforementioned scores (SNAP II, SNAPPE II, SNAPPE, modified NEOMOD) when adding EXTEM A10. The addition of A10 to SNAP II and SNAPPE II indexes apparently increased their discrimination capacity in predicting mortality (e.g., at 120 days; SNAP II, AUC point estimate: 0.768 vs. SNAP II plus A10, AUC point estimate: 0.801), though the increase was not statistically significant. Notably, the addition of A10 did not improve the performance of either SNAPPE or modified NEOMOD. These suggestive findings could be attributed to the fact that hemostatic balance is partially reflected in SNAPPE and modified NEOMOD by platelet count. Although no statistically significant difference was observed between the AUC of any univariable model and the respective multivariable model including A10, probably due to the small number of non-survivors in our study, our findings may suggest that EXTEM parameters might improve the performance of neonatal severity scores not including biomarkers of coagulopathy.

Several limitations have to be acknowledged. As disease severity scores were calculated only on the first day of disease onset along with EXTEM measurements, the dynamic changes in clinical status of critically-ill neonates over time and the potential effectiveness of clinical interventions were not evaluated and this might have influenced the prognostic power of these scores. However, this reflects the intended use of these scores, and it is noteworthy that the predictive performance of the neonatal severity scores remained stable over time. Furthermore, this is a single center study resulting in a relatively limited sample size. The number of deceased neonates is also rather small, probably inflating our chance of type-two error. On the other hand, data derived from a single center minimizes the effects of different practices on clinical approach and patient evaluation, and indirectly, on the prediction of in-hospital mortality.

In conclusion, the incorporation of thromboelastometry variables into the established neonatal severity score systems not including biomarkers of coagulopathy might contribute to the improvement of their prediction performance. The inclusion of conventional or viscoelastic hemostatic variables representing the extent of the coagulopathy seems essential for optimizing the prognostic performance of neonatal disease scoring systems. Further studies with a larger sample size are needed to confirm these findings.

## Figures and Tables

**Table 1:** Characteristics of the study population (*n* = 473).

	Mean ± SD; Median (IQR) or *n* (%)
Gender (males)	300 (63.4%)
Gestational age (weeks)	35.2 ± 4.37; 37 (32–39)
Birth weight (g)	2434 ± 983; 2580 (1480–3240)
Cesarean section	320 (67.7%)
5 min APGAR score	8.13 ± 1.54; 8 (8–9)
Temperature (°C)	36.9 ± 0.53; 37.0 (36.7–37.1)
Mean blood pressure (mmHg)	53.1 ± 9.1; 53.0 (47.0–58.0)
Blood Ph	7.38 ± 0.08; 7.39 (7.35–7.43)
SNAP II	3.87 ± 7.53; 0 (0–5)
SNAPPE II	7.86 ± 12.2; 5 (0–12)
SNAPPE	7.62 ± 10.4; 4 (1–10)
NEOMOD score	5.09 ± 2.99; 4 (3–7)
Suspected sepsis	176 (37.2%)
Sepsis	136 (28.8%)
Respiratory distress syndrome	243 (51.4%)
Intraventricular hemorrhage	147 (31.1%)
Intrauterine growth retardation	57 (12.1%)
Perinatal hypoxia	144 (30.4%)
Acute renal failure	70 (14.8%)
Disseminated intravascular coagulopathy	77 (16.3%)
Periventricular leuokomalacia	69 (14.6%)
Seizures	32 (6.8%)
Necrotizing enterocolitis	18 (3.8%)
WBC (×10^3^, cells/µL)	14.5 ± 8.07; 12.9 (9.47–17.9)
Neutrophils (%)	58.3 ± 17.5; 60.5 (47.0–72.0)
Platelets (×10^3^, cells/µL)	216 ± 136; 221 (100–295)
Albumin (g/dL)	2.56 ± 0.43; 2.50 (2.30–2.80)
Hematocrit (%)	41.1 ± 7.08; 40.9 (36.3–45.9)
C-reactive protein (mg/L)	33.1 ± 45.2; 13.6 (3.40–47.2)
SGOT (IU/L)	120 ± 397; 56.0 (34.0–92.0)
SGPT (IU/L)	47.7 ± 125; 19.0 (12.0–35.0)
Total bilirubin (mg/dL)	7.57 ± 6.63; 6.05 (3.95–9.45)
Direct bilirubin (mg/dL)	1.39 ± 4.39; 0.30 (0.20–0.50)
Blood urea nitrogen (mg/dL)	40.4 ± 35.2; 30.0 (20.0–50.0)
Creatinine (mg/dL)	0.66 ± 0.39; 0.60 (0.40–0.80)
Extem ROTEMS parameters:	
CT	76.4 ± 294; 52.0 (45.0–63.0)
A10	49.3 ± 15.3; 52.0 (41.0–60.0)
A20	54.6 ± 14.8; 58.0 (47.0–65.0)
A30	55.6 ± 14.5; 58.0 (48.0–65.0)
CFT	189 ± 521; 88.0 (62.0–140)
MCF (mm)	56.7 ± 15.0; 59.0 (49.0–67.0)
ALPHA-angle	71.5 ± 12.3; 75.0 (68.0–80.0)
LI60 (%)	94.5 ± 7.46; 95.0 (93.0–99.0)
ML (%)	10.1 ± 12.0; 8.00 (3.00–13.00)

Abbreviations: SNAP, score for neonatal acute physiology; SNAPPE, score for neonatal acute physiology with perinatal extension; NEOMOD, modified neonatal multiple organ dysfunction; SD, standard deviation; IQR, interquartile range; CT, clotting time; CFT, clot formation time; A10, clot amplitude at 10 min; A20, clot amplitude at 20 min; A30, clot amplitude at 30 min; MCF, maximum clot firmness; LI60, lysis index at 60 min; ML, maximal lysis.

**Table 2:** Area under curve (AUC) showing the prognostic power of the mortality indexes over time.

Time (Days) ^a^	SNAP II	SNAPPE II	SNAPPE	Modified NEOMOD
5	0.724 (0.617–0.830)	0.750 (0.638–0.863)	0.828 (0.741–0.915) ^b,c^	0.858 (0.779–0.936) ^b,c^
15	0.746 (0.644–0.847)	0.765 (0.659–0.871)	0.818 (0.725–0.910)	0.843 (0.757–0.929) ^b^
30	0.746 (0.656–0.837)	0.767 (0.675–0.859)	0.834 (0.756–0.912) ^b,c^	0.851 (0.780–0.921) ^b,c^
60	0.755 (0.668–0.842)	0.783 (0.697–0.869)	0.852 (0.780–0.925) ^b,c^	0.856 (0.792–0.920) ^b,c^
90	0.755 (0.668–0.842)	0.783 (0.697–0.870)	0.852 (0.780–0.925) ^b,c^	0.856 (0.791–0.920) ^b,c^
120	0.768 (0.685–0.852)	0.796 (0.713–0.879)	0.862 (0.793–0.930) ^b,c^	0.860 (0.798–0.921) ^b^

Abbreviations: SNAP, score for neonatal acute physiology; Modified NEOMOD, Neonatal Multiple Organ Dysfunction; SNAPPE, Score for Neonatal Acute Physiology and SNAP Perinatal Extension. Footnotes: ^a^ days since time zero; time zero is the time of disease onset. ^b^ the comparison of the AUC with SNAP II (at the same time point) was statistically significant (*p* < 0.05). ^c^ the comparison of the AUC with SNAPPE II (at the same time point) was statistically significant (*p* < 0.05).

**Table 3:** Area under the Curve (AUC) showing the prognostic power of mortality indexes for preterm and term infants over time.

Time (Days) ^a^	SNAP II	SNAPPE II	SNAPPE	Modified NEOMOD
Preterm neonates (*n* = 224)				
5	0.688 (0.548–0.828)	0.747 (0.595–0.900)	0.815 (0.714–0.916)	0.792 (0.664–0.921)
15	0.715 (0.580–0.850)	0.770 (0.625–0.915)	0.834 (0.737–0.930) ^b^	0.806 (0.683–0.928)
30	0.709 (0.591–0.828)	0.763 (0.641–0.885)	0.843 (0.763–0.924) ^b^	0.819 (0.720–0.917)
60	0.694 (0.574–0.814)	0.760 (0.641–0.879)	0.847 (0.768–0.925) ^b^	0.818 (0.723–0.912)
90	0.695 (0.575–0.815)	0.761 (0.642–0.880)	0.847 (0.769–0.926) ^b^	0.817 (0.722–0.912)
120	0.693 (0.574–0.813)	0.761 (0.643–0.879)	0.849 (0.771–0.927) ^b^	0.820 (0.725–0.916)
Term neonates ^d^ (*n* = 249)				
5	0.827 (0.676–0.978)	0.783 (0.612–0.954)	0.819 (0.644–0.994)	0.928 (0.855–0.999) ^b,c^
15	0.814 (0.666–0.962)	0.780 (0.617–0.944)	0.779 (0.602–0.956)	0.865 (0.728–0.999)
30	0.821 (0.688–0.954)	0.787 (0.641–0.933)	0.805 (0.657–0.952)	0.868 (0.757–0.979)
60	0.845 (0.731–0.959)	0.820 (0.693–0.947)	0.843 (0.714–0.972)	0.881 (0.787–0.975)
90	0.845 (0.731–0.959)	0.820 (0.693–0.947)	0.843 (0.714–0.972)	0.881 (0.787–0.975)

Abbreviations: SNAP, score for neonatal acute physiology; Modified NEOMOD, Neonatal Multiple Organ Dysfunction; SNAPPE, Score for Neonatal Acute Physiology and SNAP Perinatal Extension. Footnotes: ^a^ days since time zero; time zero is the time of disease onset. ^b^ the comparison of the AUC with SNAP II (at the same time point) was statistically significant (*p* < 0.05). ^c^ the comparison of the AUC with SNAPPE II (at the same time point) was statistically significant (*p* < 0.05). ^d^ note that the maximum follow-up time of term neonates was shorter; thus, we calculated the AUC until 90 days for this group.

## Data Availability

The data presented in this study are available on request from the corresponding author.
